# Impact of Histone H1 on the Progression of Allergic Rhinitis and Its Suppression by Neutralizing Antibody in Mice

**DOI:** 10.1371/journal.pone.0153630

**Published:** 2016-04-18

**Authors:** Toshiaki Nakano, Rikiya Kamei, Takashi Fujimura, Yuki Takaoka, Ayane Hori, Chia-Yun Lai, Kuei-Chen Chiang, Yayoi Shimada, Naoya Ohmori, Takeshi Goto, Kazuhisa Ono, Chao-Long Chen, Shigeru Goto, Seiji Kawamoto

**Affiliations:** 1 Graduate Institute of Clinical Medical Sciences, Chang Gung University College of Medicine, Kaohsiung, Taiwan; 2 Liver Transplantation Center and Division of Transplant Immunology, Institute for Translational Research in Biomedicine, Kaohsiung Chang Gung Memorial Hospital, Kaohsiung, Taiwan; 3 Department of Molecular Biotechnology, Graduate School of Advanced Sciences of Matter, Hiroshima University, Higashi-Hiroshima, Japan; 4 Center for Gene Science, Hiroshima University, Higashi-Hiroshima, Japan; 5 Kazusa Institute for Drug Discovery, Josai International University, Kisarazu, Japan; 6 Basic Medical Science of Nursing, Faculty of Nursing, Department of Nursing, Josai International University, Togane, Japan; 7 Department of Food Sciences and Biotechnology, Faculty of Life Sciences, Hiroshima Institute of Technology, Hiroshima, Japan; 8 Fukuoka Institution of Occupational Health, Fukuoka, Japan; Toho University School of Medicine, JAPAN

## Abstract

Nuclear antigens are known to trigger off innate and adaptive immune responses. Recent studies have found that the complex of nucleic acids and core histones that are derived from damaged cells may regulate allergic responses. However, no fundamental study has been performed concerning the role of linker histone H1 in mast cell-mediated type I hyperreactivity. In this study, we explored the impact of histone H1 on mast cell-mediated allergic responses both *in vitro* and *in vivo*. In the course of a bona-fide experimental allergen sensitization model upon co-injection with alum adjuvant, ovalbumin (OVA), but not PBS, induced elevated levels of circulating histone H1. Intranasal challenge with histone H1 to OVA/alum- (but not PBS/alum)-sensitized mice induced significantly severer symptoms of allergic rhinitis than those in mice sensitized and challenged with OVA. A monoclonal antibody against histone H1 not only suppressed mast cell degranulation, but also ameliorated OVA-induced nasal hyperreactivity and IgE-mediated passive cutaneous anaphylaxis. Our present data suggest that nuclear histone H1 represents an alarmin-like endogenous mediator acting on mast cells, and that its blockage has a therapeutic potential for mast cell-mediated type I hyperreactivity.

## Introduction

The release of nuclear antigens from damaged cells and/or activated immune cells, such as dendritic cells (DCs) and macrophages, into the blood stream has been associated with the progression of several diseases, including infectious diseases, proinflammatory disorders, malignancies, and traumas [1–5]. In previous studies, we have demonstrated elevated levels of circulating histone H1 and high-mobility group box 1 (HMGB1) in the course of liver transplant rejection [6,7]. These nuclear antigens may act as alarmins in the induction of proinflammatory immune responses. Thus, prevention of those nuclear antigen-driven innate immune signals would be a therapeutic target for proinflammatory disorders. We have demonstrated the transient induction of autoantibody (auto-Abs) against histone H1 and HMGB1, which may be related to long-term liver allograft acceptance [8–11]. The therapeutic potential of anti-histone H1 Ab or anti-HMGB1 Ab has been demonstrated in experimental sepsis [12–14], liver ischemia/reperfusion injury [15], Concanavalin A-induced hepatic injury [16,17], traumatic brain injury [18], and organ transplantation [8–11,19,20]. Our *in vitro* studies have also elucidated the unique role of nuclear proteins and their Abs in both individual cells and cell-to-cell interactions among DCs, T cells, and mesenchymal stem cells [3,21,22]. Recently, we demonstrated that mast cells, which usually induce allergic reactions, are involved in immunological tolerance through the induction of regulatory T cells and liver regeneration by mast cell activation in the course of rejection and following tolerance induction [23]. Transient elevation of hepatic histone H1 and circulating anti-histone H1 Ab in the course of rejection is one of important events for overcoming rejection [24], and these data strongly suggest the involvement of histone H1 and corresponding Ab for regulation of mast cell activity in transplant immunology. To further explore underlying mechanism regarding histone H1 and corresponding Ab in mast cells, we shift our focus on mast cell-mediated allergic response in the present study.

Allergic rhinitis, which includes pollinosis, is categorized as a type I hyperreactivity and depends on the interaction between antigens and the antigen-specific IgE Ab attached to mast cells [25]. Recently, several studies have detected the induction of nasal HMGB1 in allergic rhinitis [26], chronic rhinosinusitis [27], and upper airway inflammatory diseases [28], suggesting the involvement of nuclear antigens in the induction of allergic responses. In the course of a bona-fide experimental allergen sensitization model upon co-injection with alum adjuvant, the alum causes cell death and the subsequent release of host cell DNA mediates allergen-specific Th2 response and IgE production [29]. A recent paper also noted the significance of host cell DNA complexed with core histones, but not linker histone H1, in the initiation of a T cell-intrinsic Th2 cell differentiation by unknown innate immune mechanisms [30]. However, it is unclear about the role of histone H1 in mast cell-mediated type I hyperreactivity.

In this study, we show impact of endogenous linker histone H1 on the progression of allergic rhinitis-like nasal symptoms in mice as well as on its positive regulatory role in mast cell degranulation. The therapeutic potential of a newly developed monoclonal Ab (mAb) against a histone H1 peptide mimotope (SSVLYGGPPSAA) referred to as SSV mAb, which is responsible for the immunosuppression of anti-histone H1 mAb [31], was also evaluated.

## Materials and Methods

### Ethics statement

Our experimental design was reviewed and approved by the Institutional Animal Care and Use Committee in Kaohsiung Chang Gung Memorial Hospital (approval No.: 2014101601). The Committee recognizes that the proposed animal experiment follows the Animal Protection Law by the Council of Agriculture, Executive Yuan, R.O.C. and the guideline shown in the Guide for the Care and Use of Laboratory Animals, as promulgated by the Institute of Laboratory Animal Resources, National Research Council, USA.

### Animals

Male Lewis rats and female BALB/c mice were obtained at 5 weeks of age from the National Laboratory Animal Breeding and Research Center (Taipei, Taiwan) or Charles River Laboratories (Yokohama, Japan). All animals were maintained under specific pathogen-free animal facilities with water and commercial diet *ad libitum*. All serum samples were stored at -80°C until analysis.

### Preparation of anti-histone H1 peptide mAb

The SSV hybridoma, which produces an immunosuppressive mAb against a histone H1 peptide mimotope (SSVLYGGPPSAA) referred to as SSV mAb, was generated by Josai International University, Japan, and its specific binding to histone H1 was demonstrated [14]. The SSV hybridoma (isotype: IgG1) was cultured for 1 week in RPMI-1640 medium (Invitrogen, Carlsbad, CA, USA) supplemented with penicillin-streptomycin (Invitrogen) and 15% fetal bovine serum (FBS, Invitrogen) at 37°C. The culture supernatants were harvested, and the proteins were precipitated by 40% ammonium sulfate. The precipitate was then dissolved in PBS and dialyzed against PBS at 4°C overnight. SSV mAb was purified using a HiTrap^™^ NHS-activated column (GE Healthcare Bio-Sciences Corp., Piscataway, NJ, USA) coupled with the histone H1 peptide mimotope (SSVLYGGPPSAA) conjugated to keyhole limpet hemocyanin.

### Ovalbumin (OVA)-induced allergic rhinitis model

Immunization and nasal challenge with OVA (Sigma, St. Louis, MO, USA) were performed by previously described protocols [32,33] with some modification. Briefly, BALB/c mice (n = 21) were intraperitoneally immunized twice with a precipitate (100 μl/mouse) of alum (2 mg, Thermo Fisher Scientific Inc., Rockford, IL, USA) and OVA (20 μg), with a two-week interval. After the second immunization, calf thymus linker histone H1 (20 mg/ml, 20 μl/mouse, n = 4, Merck Millipore, Billerica, MA, USA), PBS (negative control: 20 μl/mouse, n = 4) or OVA (positive control: 20 mg/ml, 20 μl/mouse, n = 4) was intranasally administered to evaluate the fundamental role of histone H1 in allergic rhinitis. As a control for OVA/alum sensitization, BALB/c mice (n = 6) were intraperitoneally immunized twice with a precipitate (100 μl/mouse) of alum (2 mg, Thermo Fisher Scientific Inc.) and PBS, followed by the intranasal administration of calf thymus histone H1 (20 mg/ml, 20 μl/mouse, n = 3) or PBS (negative control: 20 μl/mouse, n = 3). To evaluate the therapeutic significance of histone H1-targeted SSV mAb, 100 μg of SSV mAb (n = 4) or isotype IgG1 (n = 5) was intraperitoneally administered before the daily nasal challenge with OVA (50 mg/ml, 20 μl/mouse). To evaluate the allergic rhinitis-like symptoms, the number of sneezing and nasal rubbing were counted for 5 minutes after the histone H1 or OVA nasal challenge.

### Quantification of nuclear histone H1

The histone H1 levels in the serum before and after OVA sensitization or after final nasal challenge with OVA were determined using an enzyme-linked immunosorbent assay (ELISA), as described previously [6,7].

### OVA-specific immunoglobulins

The serum levels of OVA-specific IgG1, IgG2a and IgE were determined by ELISA as described previously [33]. Briefly, OVA solution (50 mg/ml, in PBS) was coated onto 96-well microplates (Nalgene Nunc International, Roskilde, Denmark) and incubated overnight at 4°C. The plate was then blocked with 1% (w/v) bovine serum albumin (BSA)/PBS for 2 hrs at 37°C, and serum samples (IgG1: 10,000× dilution, IgG2a: 1,000× dilution, IgE: 10× dilution with blocking buffer) were added to each well and incubated for 2 hrs at 37°C. Next, biotin-conjugated anti-mouse IgG1, IgG2a or IgE (250× dilution; BD Biosciences, San Jose, CA, USA) was added and incubated at 37°C for 1.5 hrs. Streptavidin-HRP (200× dilution; R&D Systems, Minneapolis, MN, USA) was then added and incubated at 37°C for 1 hr, followed by the addition of 1-Step Ultra TMB substrate solution (Thermo Fisher Scientific Inc.). The absorbance at 450 nm was then measured using a Victor X4 Multilabel Plate Reader (PerkinElmer, Waltham, MA, USA).

### Histological analysis and immunohistochemical (IHC) staining

Experimental mice were sacrificed after the final OVA challenge, and the heads were kept in 10% formaldehyde solution. Decalcification was performed by previously described protocol [34]. The specimens were embedded in paraffin, and nasal sections (4 μm) were stained with hematoxylin and eosin (H&E) or toluidine blue to assess inflammatory cell infiltration. Eosinophils and mast cells were counted under a microscope at ×400 magnification. At least six randomly chosen high power fields (HPFs) of each section were examined to yield the mean number of eosinophils or mast cells per HPF in the nasal septal mucosa.

For IHC staining, dewaxed slides were incubated with Peroxidase Block (3% H_2_O_2_) for 20 min and heated in sodium citrate buffer (pH 6.0) at 95°C for 20 min. After washing and blocking, the slides were incubated with rabbit polyclonal Ab against histone H1 (100× dilution; Santa Cruz Biotechnology, Santa Cruz, CA, USA) for 2 hrs followed by incubation with Mouse/Rabbit Probe HRP labeling (BioTnA, Kaohsiung, Taiwan) for 30 min. After washing, chromogen development was performed by 3,3’-diaminobenzidine staining. Counter staining was carried out using hematoxylin. The slides were rinsed with H_2_O and covered with resin-based mounting medium (BioTnA) after dehydration.

### Mast cell degranulation

Rat basophilic leukemia cell line RBL-2H3 and Lewis bone marrow-derived mast cells (BMMCs) [23] were used in this study. Briefly, RBL-2H3 or BMMCs (1×10^6^ cells/ml) were sensitized with anti-DNP IgE (0.5 μg/ml; Sigma) for 18 hrs at 37°C. They were then washed with Tyrode’s buffer (10 mM HEPES, 130 mM NaCl, 5 mM KCl, 1.4 mM CaCl_2_, 1 mM MgCl_2_, 5.6 mM glucose, 0.1% BSA, pH 7.4), and 2.5×10^6^ cells/ml of RBL-2H3 or BMMCs were incubated in the presence of calf thymus histone H1 (25, 50 and 100 μg/ml; Merck Millipore), SSV mAb (25, 50 and 100 μg/ml) or IgG1 isotype control (100 μg/ml; BD Biosciences) for 1 hr at 37°C. To induce degranulation, 0.5 μg/ml of DNP-BSA (Invitrogen) was added. The supernatants were collected, and 0.5% Triton X-100 solution in Tyrode’s buffer was added to quantify the enzyme activity remaining in the cells. To quantify the β-hexosaminidase activity, 20 μl of substrate solution (1.3 mg/ml *p*-nitrophenyl-N-acetyl-β-D-glucosaminide in 0.04 M sodium citrate, pH 4.5) was added to each sample (30 μl) and incubated for 1 hr at 37°C under gentle rotation. To stop the enzymatic reaction, 150 μl of stop solution (0.2 M glycine, pH 10.0) was added, and the absorbance at 405 nm was determined using a Victor X4 Multilabel Plate Reader (PerkinElmer). The percentage of degranulation was calculated using the following formula: Degranulation rate (%) = 100 × absorbance of supernatant/(absorbance of supernatant + absorbance of cell lysate).

### Western blot analysis for extracellular histone H1

Calf thymus histone H1 (10 μg; Merck Millipore) and culture supernatants (5 μl) of BMMCs with 0.1 or 0.5 μg/ml of DNP-BSA (Invitrogen) stimulation and were run on a 12.5% SDS-PAGE gel using a mini gel apparatus (Bio-Rad, Burlington, MA, USA), and fractionated proteins were electronically transferred onto a PVDF membrane (GE Healthcare Bio-Sciences Corp.). The membrane was blocked using 5% skim milk at room temperature for 1 hr and immunoprobed with rabbit polyclonal Ab against histone H1 (200× dilution with 5% skim milk/PBST; Santa Cruz Biotechnology) followed by incubation with peroxidase-conjugated goat anti-rabbit IgG (10,000× dilution; Santa Cruz Biotechnology). Signals were visualized using an ECL Plus Western Blotting Detection System (GE Healthcare Bio-Sciences Corp.).

### Passive cutaneous anaphylaxis (PCA) model

BALB/c mice were used to evaluate PCA response according to previously described protocols [35,36] with some modification. Briefly, anti-DNP IgE (150 ng/10 μl; Sigma) was injected intradermally in the right ear. As a blank, PBS was injected intradermally in the left ear. To explore the effect of histone H1 on PCA response, calf thymus histone H1 (5 μg/10 μl; Merck Millipore) was injected intradermally both in the left and right ears (n = 3). As a sham control (n = 8), PBS was injected intradermally in both right and left ears. Twenty-three hrs later, 200 μl of PBS (vehicle control; n = 7), 100 μg of isotype IgG1 (n = 8) or SSV mAb (n = 7) was injected intravenously via tail vein. One hr later, 200 μg of DNP-HSA (Sigma) in 100 μl of evans blue solution (0.5 or 1%; Tokyo Chemical Industry, Co., Ltd., Tokyo, Japan) was injected intravenously to induce anaphylaxis. After 30 minutes, PCA response was evaluated by the measurement of evans blue dye in the ears. Evans blue dye in the ears was extracted by overnight incubation with formamide at 63°C, and the absorbance at 595 nm was determined using a Wallac 1420 ARVOsx Multilabel Counter (PerkinElmer). This study was performed in Hiroshima University using protocols reviewed and approved by the under the Committee on Animal Experimentation of Hiroshima University.

### Cytokine production in splenocytes

Splenocytes from mice after final OVA nasal challenge were cultured for 72 hrs at 37°C in the presence of OVA (200 μg/ml) in RPMI-1640 medium (Invitrogen) supplemented with penicillin-streptomycin (Invitrogen) and 10% FBS (Invitrogen). The supernatants were then collected, and the IFN-γ, IL-4, IL-5, IL-10, IL-13, and IL-17 levels were measured using DuoSet ELISA Development Kits (R&D Systems), according to the manufacturer’s protocols.

### Statistical analysis

Unpaired two-tailed Student’s *t*-tests were used to determine the significance of the difference between the normally distributed means of value in the two groups. Each sample was tested in triplicate, and the results are indicated as the mean ± S.D.

## Results

### Elevation of circulating histone H1 by allergen sensitization

**Fig 1:**
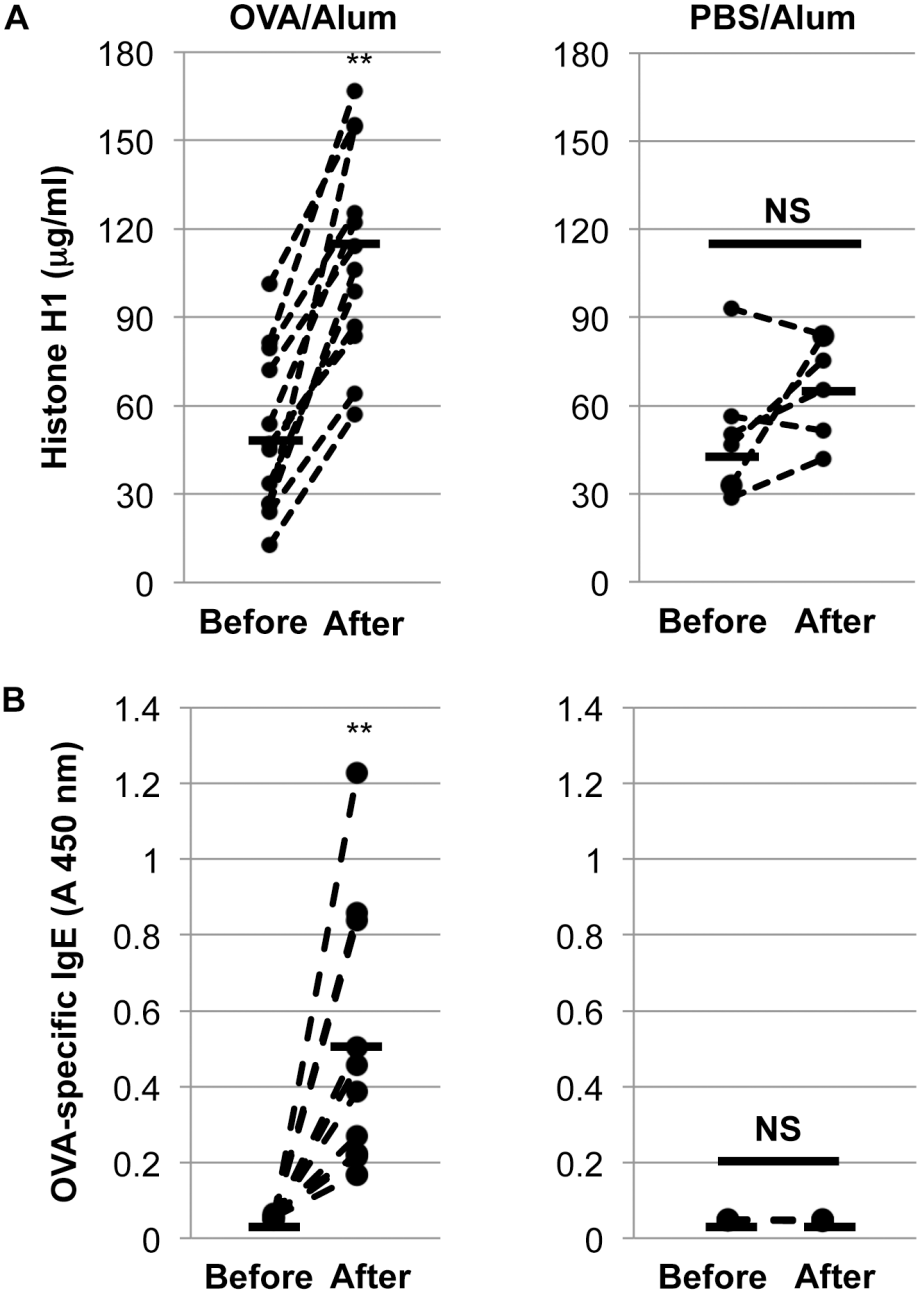
Induction of circulating histone H1 by OVA/alum sensitization and its correlation with OVA-specific IgE production. Circulating histone H1 (A) and OVA-specific IgE (B) levels were measured by ELISA. **, *P*<0.01 versus the pre-immunized serum. NS: not significant.

To explore the fundamental role of histone H1 in the course of allergen sensitization *in vivo*, the serum levels of histone H1 before and after OVA sensitization were evaluated. As shown in Fig 1A, OVA/alum-sensitized mice (n = 12) expressed significantly higher levels of histone H1 in the serum. The total (S1 Fig) and OVA-specific IgE levels were also elevated after OVA/alum sensitization (Fig 1B). However, PBS/alum sensitization (n = 6) failed to elevate either the histone H1 or total/OVA-specific IgE levels (Fig 1A and 1B). These results suggest the significance of OVA/alum sensitization in the activation of proinflammatory immune responses, resulting in the elevation of both the total/OVA-specific IgE and circulating histone H1 levels *in vivo*.

### Induction of allergic rhinitis by histone H1 in OVA-sensitized mice

**Fig 2:**
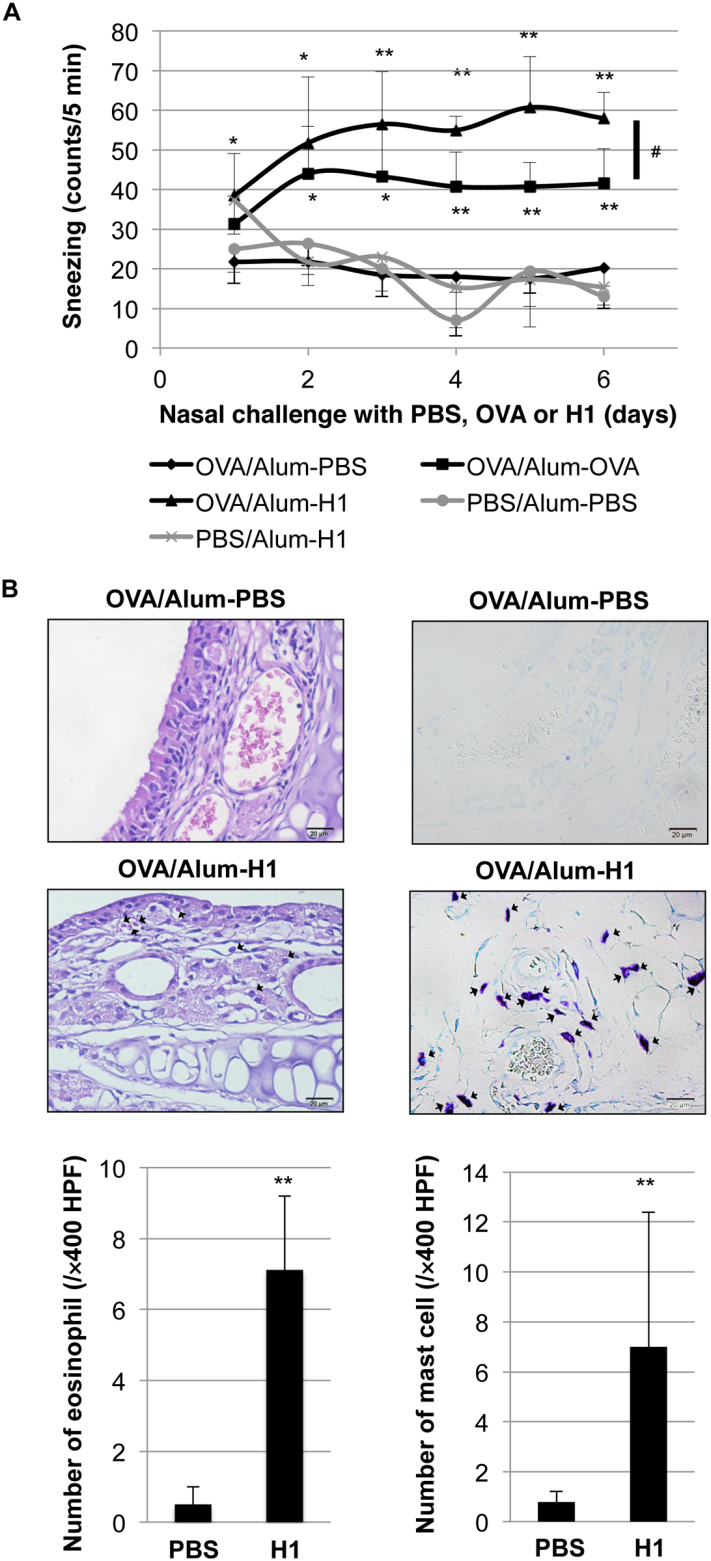
Induction of allergic rhinitis-like symptoms by intranasal challenge with histone H1 in OVA/alum-sensitized mice. (A) The instance of sneezing was counted for 5 minutes immediately after calf thymus histone H1 (0.4 mg), OVA (0.4 mg) or PBS nasal challenge. *, *P*<0.05, **, *P*<0.01 versus the OVA/alum-sensitized and PBS-challenged group. ^#^, *P*<0.05 versus the OVA/alum-sensitized and OVA-challenged group. (B) Representative sections of the nasal septal mucosa in OVA/alum-sensitized and PBS or histone H1-challenged group (n = 4 per group). Eosinophils (left: H&E staining) and mast cells (right: toluidine blue staining) (shown by arrows) were counted under a microscope at ×400 magnification. Values are presented as the mean ± S.D. **, *P*<0.01 versus the OVA/alum-sensitized and PBS-challenged group.

To explore the pathophysiological role of OVA/alum sensitization-induced histone H1 in the model of allergic rhinitis, a daily intranasal administration of calf thymus histone H1 (0.4 mg) was performed after OVA/alum sensitization. As shown in Fig 2A, histone H1 significantly induced symptoms of allergic rhinitis such as sneezing and nasal rubbing (data not shown), and the frequency of sneezing was higher after histone H1 challenge than it was after OVA (0.4 mg) challenge. However, histone H1 failed to induce allergic rhinitis in PBS/alum-sensitized mice, suggesting the involvement of histone H1 in mast cell-mediated allergic responses in the presence of elevating total/OVA-specific IgE. Pathological evidence demonstrated the damage of nasal epithelium and infiltration of eosinophils and mast cells by histone H1 as compared with negative control PBS (Fig 2B).

### Enhancement of degranulation by histone H1 and suppression of degranulation by corresponding SSV mAb

**Fig 3:**
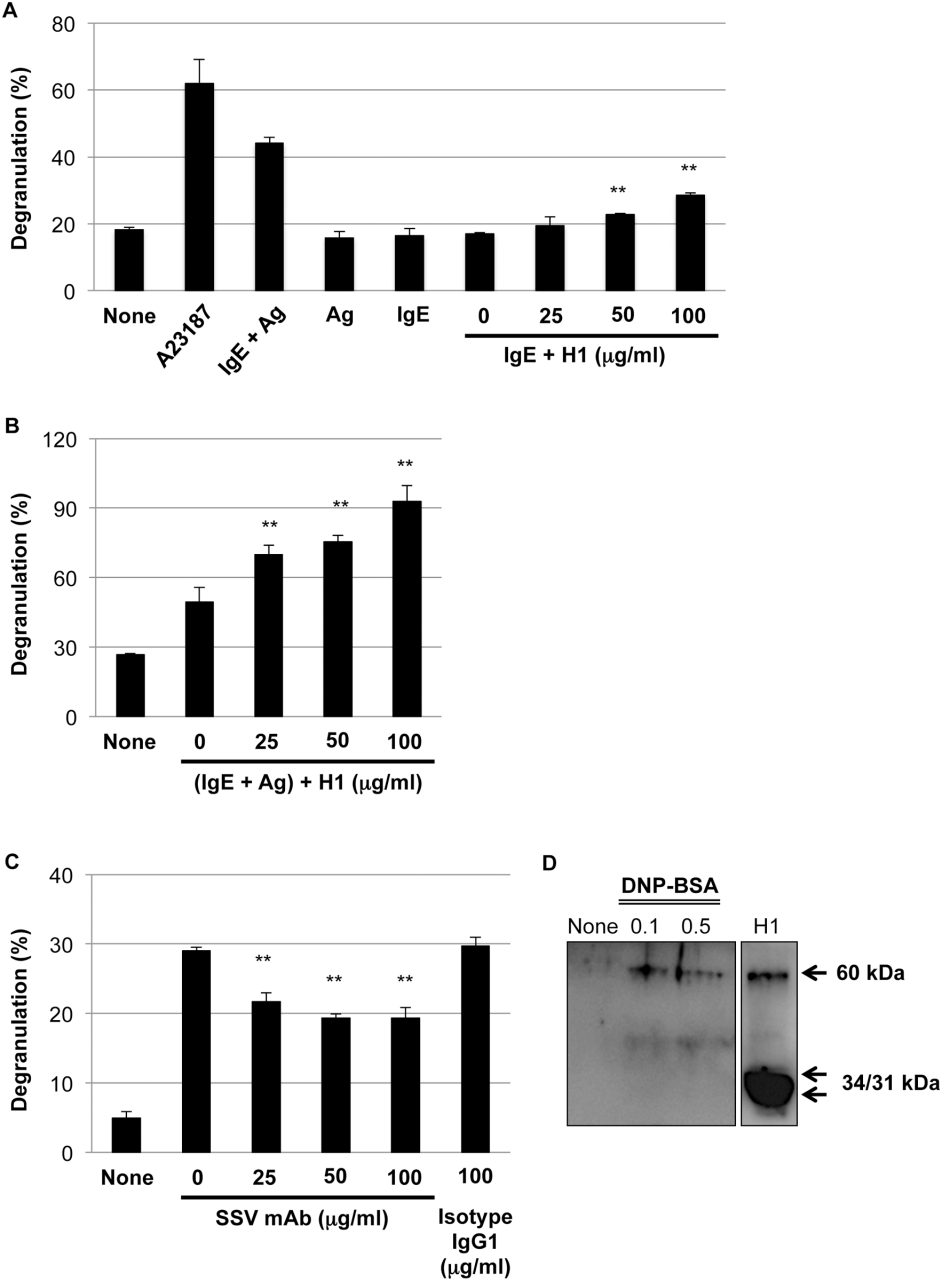
Histone H1-mediated mast cell degranulation. (A) Exogenous histone H1 dose-dependently induced degranulation of IgE-sensitized rat basophilic leukemia cell line RBL-2H3 without cross-linking between anti-DNP-IgE and antigen (Ag: DNP-BSA). Degranulation was evaluated by β-hexosaminidase release rate. Values are presented as the mean ± S.D. from three independent experiments. A23187 (10 μM) was used as a positive control for degranulation. **, *P* < 0.01 versus IgE only (0 μg/ml of histone H1). (B) Exogenous histone H1 enhanced DNP-BSA-induced degranulation of RBL-2H3 cells in a dose-dependent manner. **, *P* < 0.01 versus (IgE + Ag: 0 μg/ml of histone H1). (C) Histone H1-targeted SSV mAb ameliorated DNP-BSA-induced degranulation of RBL-2H3 cells in a dose-dependent manner. **, *P* < 0.01 versus (IgE + Ag: 0 μg/ml of SSV mAb) or (IgE + Ag: 100 μg/ml of isotype IgG1). (D) The extracellular secretion of endogenous histone H1 by DNP-BSA (0.1 and 0.5 μg/ml) in BMMCs. Western blot data are representative of three independent experiments. Calf thymus histone H1 was loaded as a positive control.

To demonstrate the pathophysiological roles of histone H1 on mast cell-mediated allergic responses *in vitro*, rat basophilic leukemia cell line RBL-2H3 were sensitized with anti-DNP IgE and then stimulated with calf thymus histone H1 (25, 50 and 100 μg/ml) in the absence of DNP-BSA. As shown in Fig 3A, exogenous histone H1 significantly induced the degranulation without cross-linking in a dose-dependent manner. Furthermore, histone H1 enhanced DNP-BSA-induced degranulation in a dose-dependent manner (Fig 3B). To further explore the role of histone H1 and therapeutic potential of histone H1-targeted SSV mAb on degranulation, rat RBL-2H3 were sensitized with anti-DNP IgE and then treated with SSV mAb (25, 50 and 100 μg/ml) or isotype IgG1 (100 μg/ml) under stimulation with DNP-BSA. Similar data were also obtained by using BMMCs (data not shown). As shown in Fig 3C, DNP-BSA-induced degranulation was significantly suppressed by SSV mAb. The extracellular secretion of endogenous histone H1 by DNP-BSA in BMMCs suggested the autocrine/paracrine effects of histone H1 in the course of degranulation (Fig 3D).

### Enhancement of PCA by histone H1 and inhibition of PCA by corresponding SSV mAb

**Fig 4:**
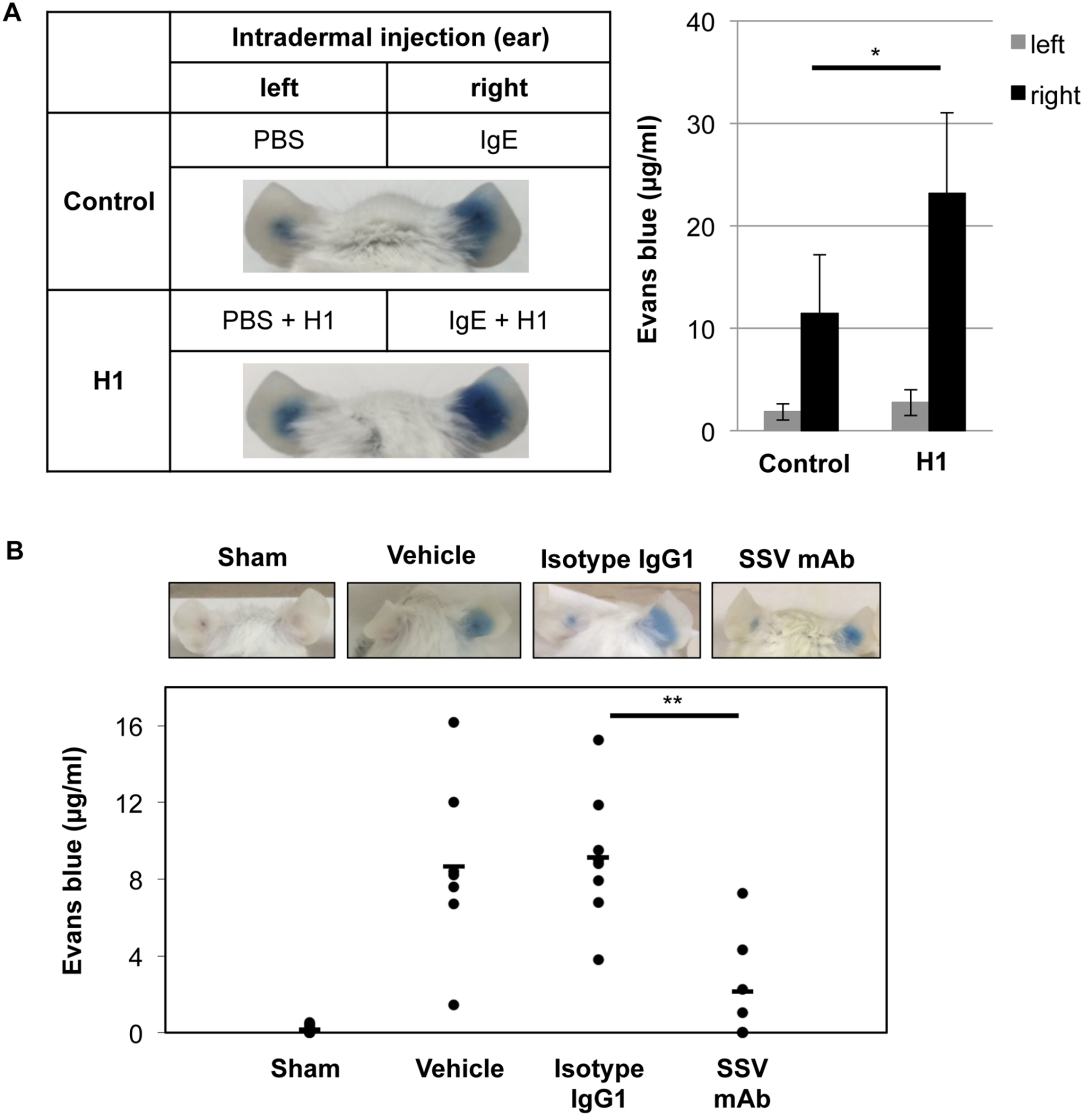
Enhancement and inhibition of IgE-mediated PCA reaction by histone H1 and histone H1-targeted SSV mAb. (A) PBS and anti-DNP IgE (150 ng/10 μl) with/without calf thymus histone H1 (5 μg) were intradermally injected into the left and right ears, respectively (n = 3 per group). After 24 hrs, DNP-HSA (200 μg) with evans blue solution (1%) was injected intravenously via tail vein to induce anaphylaxis. After 30 minutes, evans blue extravasation in the ears was observed. The pictures are representative of three individuals in each group. Data are representative of three independent experiments and represented as the mean ± S.D. *, *P*<0.05 versus the control group. (B) PBS and anti-DNP IgE (150 ng/10 μl) were intradermally injected into the left and right ears, respectively. As a sham control (n = 8), PBS was injected intradermally in both left and right ears. After 23 hrs, PBS (vehicle; n = 7), isotype IgG1 (n = 8) or SSV mAb (n = 7) was intravenously injected via tail vein. One hr later, DNP-HSA (200 μg) with evans blue solution (0.5%) was injected intravenously via tail vein to induce anaphylaxis. After 30 minutes, evans blue extravasation in the ears was observed. The pictures are representative of seven to eight individuals in each group. Each symbol indicates an individual mouse, and bars show the mean values. **, *P*<0.01 versus the isotype IgG1-injected group.

To explore the impact of histone H1 on PCA response *in vivo*, histone H1 (5 μg/10 μl) was injected intradermally both in the left and right ears. IgE-mediated PCA response induced by DNP-HSA was evaluated by the measurement of evans blue dye in the ears. As shown in Fig 4A, histone H1 without IgE sensitization (left ear) failed to induce PCA response. On the other hand, histone H1 with IgE sensitization (right ear) enhanced PCA response as compared with control group, suggesting the involvement of IgE sensitization for histone H1-mediated type I hyperreactivity. To elucidate anti-allergic mechanism of histone H1-targeted SSV mAb *in vivo*, SSV mAb or IgG1 isotype control (100 μg/mouse) was intravenously injected into the anti-DNP IgE-treated mice. As shown in Fig 4B, SSV mAb significantly suppressed the extravasation of evans blue compared with vehicle and isotype controls. Taken together, histone H1-targeted SSV mAb suppressed histone H1-mediated mast cell degranulation *in vivo*.

### Inhibition of allergic rhinitis by SSV mAb in OVA-sensitized and OVA-challenged mice

**Fig 5:**
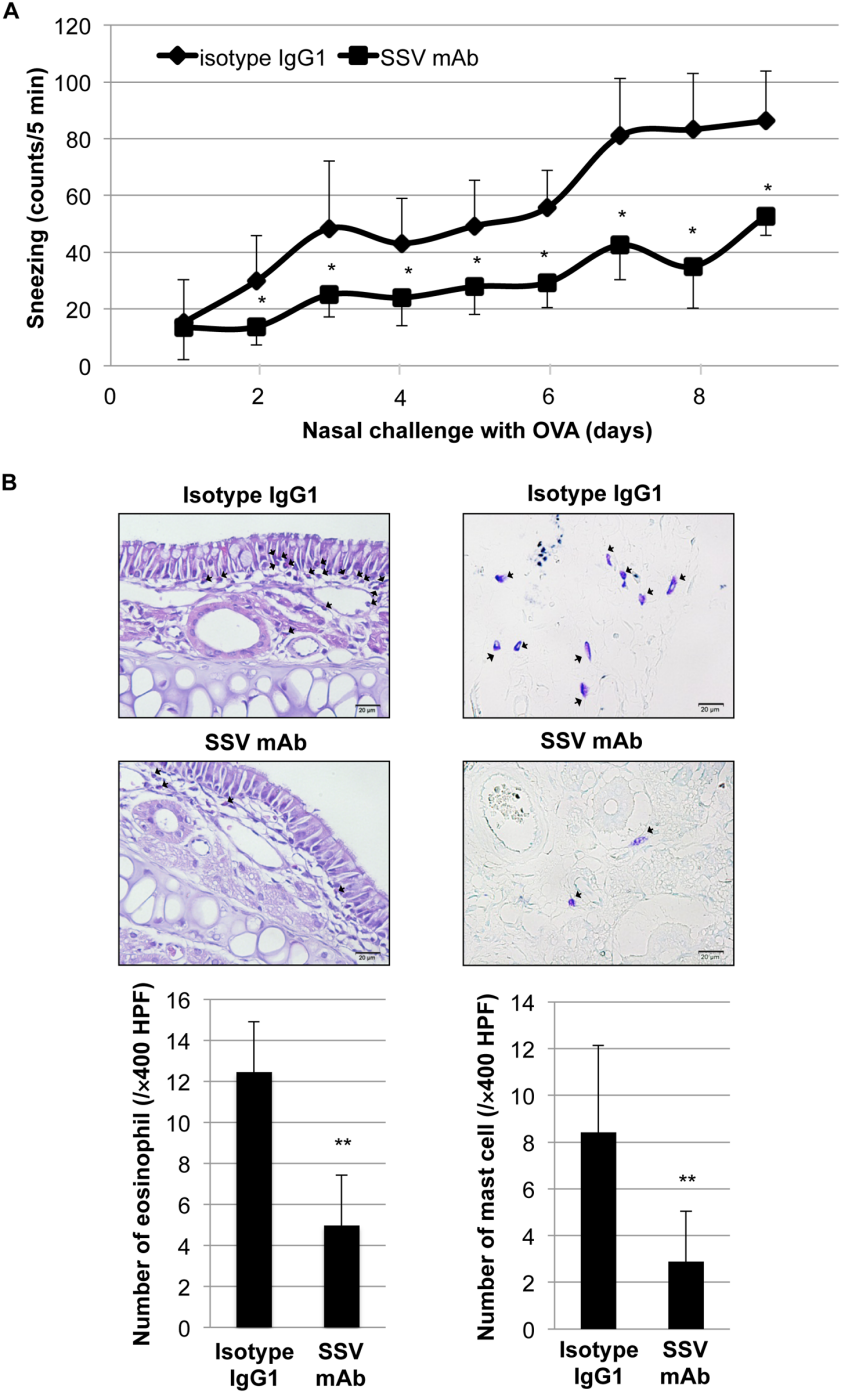
Therapeutic potential of histone H1-targeted SSV mAb on allergic rhinitis-like symptoms. Mice were immunized with OVA/alum (n = 9). SSV mAb (100 μg, n = 4) or isotype IgG1 (100 μg, n = 5) was then intraperitoneally administered before daily OVA nasal challenge. To evaluate allergic rhinitis-like symptoms, the instances of sneezing (A) and nasal rubbing (data not shown) were counted for 5 minutes immediately after OVA nasal challenge. *, *P*<0.05 versus the isotype IgG1-injected group. (B) Representative sections of the nasal septal mucosa in isotype IgG1 or SSV mAb-injected group. Eosinophils (left: H&E staining) and mast cells (right: toluidine blue staining) (shown by arrows) were counted under a microscope at ×400 magnification. Values are presented as the mean ± S.D. **, *P*<0.01 versus the isotype IgG1-injected group.

To further evaluate the therapeutic effect of SSV mAb on mast cell-mediated allergic responses *in vivo*, SSV mAb or IgG1 isotype control (100 μg/mouse) was intraperitoneally injected into the OVA/alum-sensitized mice before the daily nasal challenge with OVA (1 mg). As shown in Fig 5A, SSV mAb significantly ameliorated the symptoms of OVA-induced sneezing and nasal rubbing (data not shown) compared with the isotype control. Pathological observation demonstrated the infiltration of eosinophils and mast cells in the nasal mucosa of isotype control group, while SSV mAb markedly reduced those infiltration (Fig 5B).

### Effects of SSV mAb on OVA-specific immunoglobulins and on cytokine release from OVA-stimulated splenocytes

**Fig 6:**
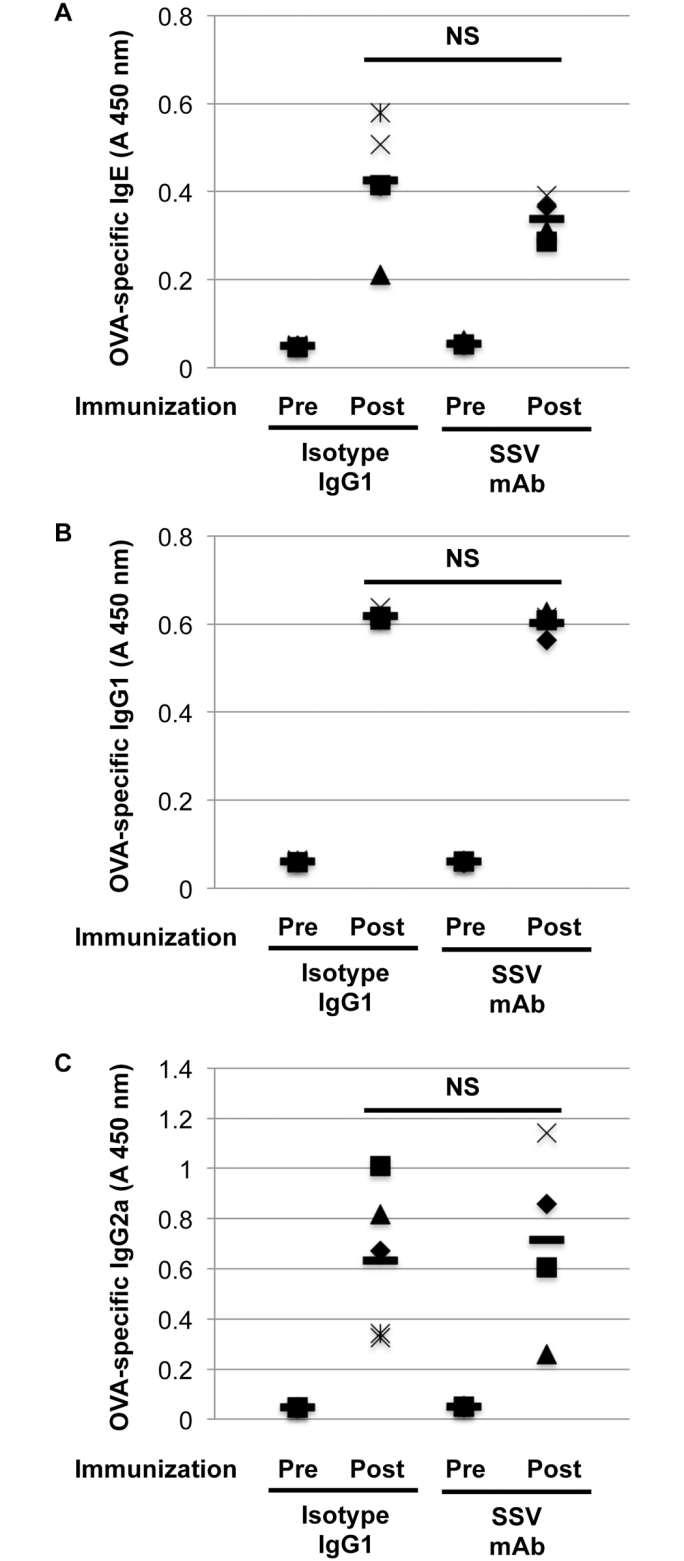
Serum OVA-specific antibody titer. Peripheral blood was obtained before OVA immunization and after final OVA nasal challenge, and serum was collected (SSV mAb (n = 4) or isotype IgG1 (n = 5)). Serum OVA-specific IgE (A), IgG1 (B) and IgG2a (C) titers were determined by ELISA. Each symbol indicates an individual mouse, and bars show the mean values. NS: not significant.

To explore the therapeutic impact of SSV mAb on OVA-specific immune responses, we determined the OVA-specific immunoglobulin levels before immunization and after the final nasal challenge. As shown in Fig 6, there was no significant effect of SSV mAb on OVA-specific IgG1, IgG2a, and IgE responses.

**Fig 7:**
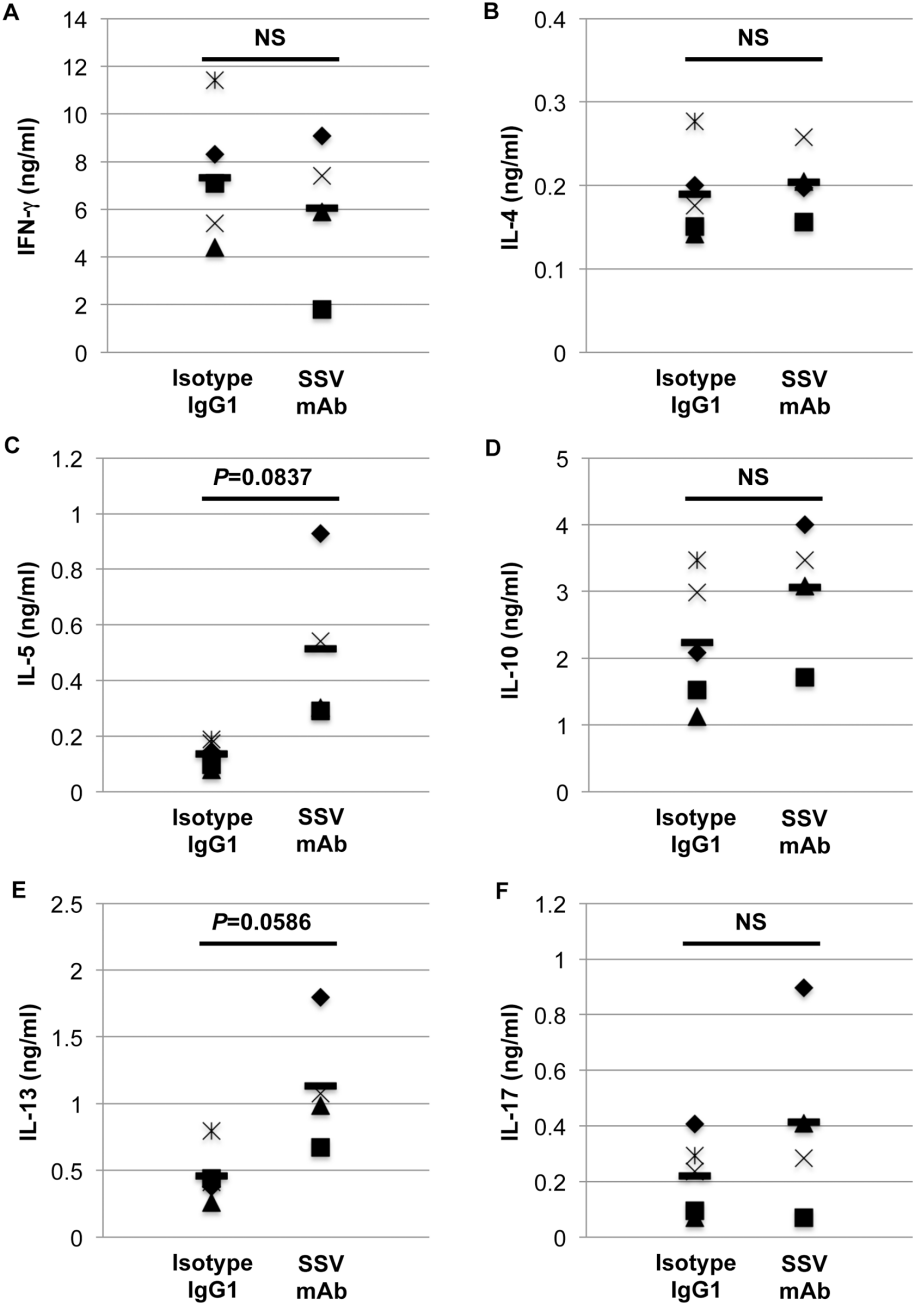
Cytokine release from total splenocytes stimulated with OVA. After the final OVA nasal challenge, the mice were sacrificed, and splenocytes were collected (SSV mAb (n = 4) or isotype IgG1 (n = 5)). Splenocytes were stimulated with OVA for 72 hrs, and culture media were obtained. Cytokine levels (A: IFN-γ, B: IL-4, C: IL-5, D: IL-10, E: IL-13, and F: IL-17) were determined by using specific ELISA kits. Each symbol indicates an individual mouse, and bars show the mean values. NS: not significant.

To explore the effect of SSV mAb on cytokine profiles, we next evaluated cytokine secretion after the OVA stimulation of splenocytes from OVA-sensitized and OVA-challenged mice. As shown in Fig 7, the IL-5 and IL-13 levels were slightly elevated by SSV mAb treatment, while the IFN-γ, IL-4, IL-10, and IL-17 levels showed no significant difference between SSV mAb- and isotype IgG1-treated mice.

Taken together, SSV mAb may minorly affect T and B cell-mediated immune responses in the course of allergic response, while it may primarily regulate mast cell-mediated type I hyperreactivity through the neutralization of histone H1.

### Impact of SSV mAb on circulating and local levels of histone H1 in OVA-sensitized and OVA-challenged mice

**Fig 8:**
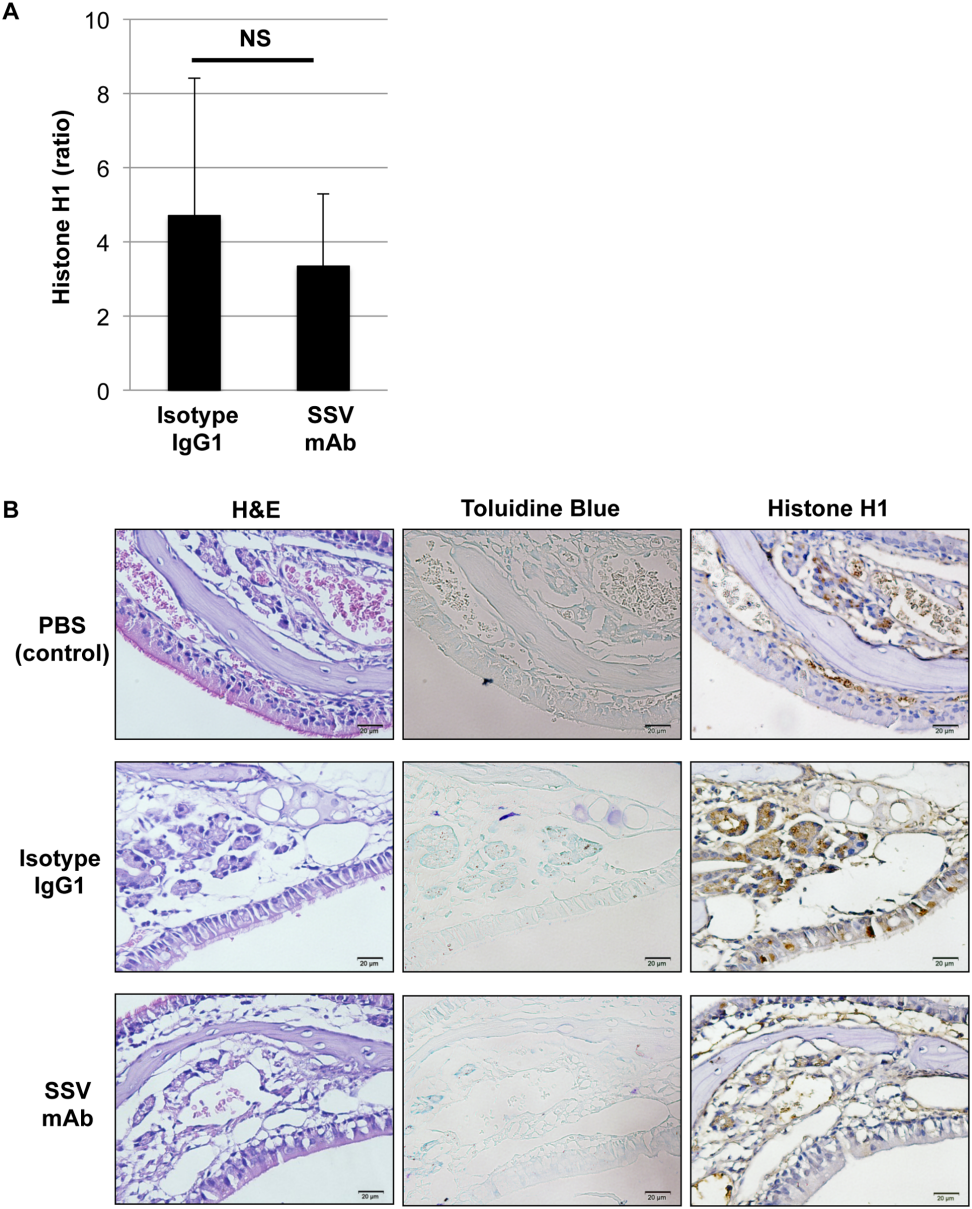
Effects of histone H1-targeted SSV mAb on circulating and local levels of histone H1. (A) Circulating histone H1 levels before nasal challenge (after 2^nd^ OVA sensitization) and after final nasal challenge with OVA were measured by ELISA. Data are represented as the mean induction ratio after nasal challenge ± S.D. NS: not significant. (B) Local expression of histone H1 in the nasal mucosa was detected by IHC staining. Eosinophils (H&E staining), mast cells (toluidine blue staining) and histone H1 (IHC staining) were detected in the serial sections (×400 magnification). Histone H1 was detected in the granules of eosinophils and mast cells located in the epithelial zone of nasal mucosa in isotype IgG1 group, while SSV mAb significantly suppressed the local expression of histone H1 in the nasal mucosa.

To explore whether SSV mAb affect the circulating level of histone H1 in OVA-sensitized and OVA-challenged mice, serum level of histone H1 was evaluated after final nasal challenge with OVA. As shown in Fig 8A, the induction ratio of circulating histone H1 after final nasal challenge as compared with 2^nd^ OVA sensitization was no obvious difference between isotype IgG1 and SSV mAb groups. On the other hand, histone H1 was detected in the granules of eosinophils and mast cells located in the epithelial zone of nasal mucosa in isotype IgG1 group, while SSV mAb significantly suppressed the local expression of histone H1 in the nasal mucosa (Fig 8B).

## Discussion

The immunological role of nuclear antigens in allergic responses is not yet fully understood. In the present study, we report for the first time the impact of linker histone H1 on the pathogenesis of mast cell-mediated allergic responses and the potential of histone H1 as a molecular target for therapeutics for mast cell-mediated type I hyperreactivity.

In our present study, we have confirmed the elevation of the circulating histone H1 levels and the total and OVA-specific IgE levels by OVA/alum sensitization (S1 Fig and Fig 1). Under this condition, an intranasal challenge of histone H1 dramatically induced the symptoms of allergic rhinitis (Fig 2). The degranulation of mast cells is a primary cause of rhinitis symptoms, and it is initiated by the interaction of antigens and the antigen-specific IgE Ab attached to mast cells. OVA/alum sensitization induced the elevation of both histone H1 and total IgE, while no definite change of histone H1-specific IgE was observed before and after OVA sensitization (S1 Fig). Therefore, histone H1 may directly affect mast cell function in OVA/alum-sensitized and histone H1-challenged mice. In our *in vitro* model of mast cell degranulation, exogenous histone H1 induced mast cell degranulation without DNP-BSA stimulation and further enhanced DNP-BSA-induced degranulation by exogenous histone H1 in a dose-dependent manner (Fig 3). Of note, IgE sensitization may be indispensable for histone H1-mediated induction of mast cell degranulation due to the lack of allergic rhinitis in PBS/Alum-sensitized and histone H1-challenged mice (Fig 2A) and the lack of PCA response in PBS/histone H1-injected ear (Fig 4A). The most probable mechanism of mast cell degranulation by histone H1 must be the autocrine/paracrine effects that histone H1 has on mast cell activation. In our previous study, we demonstrated the induction of DC maturation by exogenous histone H1 and the release of endogenous histone H1 in the course of DC maturation induced by LPS [3]. In our *in vitro* model of mast cell degranulation, exogenous histone H1 strongly induced proinflammatory cytokine release as compared with antigen-IgE cross-linking (S2 Fig). Histone H1 may be one of alarmins associated with broad range of inflammatory responses such as DC maturation [3] and IgE-mediated type I allergy shown in this study. Interestingly, the molecular weight of extracellular histone H1 in the culture supernatants of BMMCs was around 60 kDa. Similar to our observation, a previous study also suggested that extracellular histone H1 mainly exists as a dimer in the cultured live cerebellar neurons [37]. Indeed, our western blotting and CBB staining clearly indicated the existence of histone H1 with higher molecular weight (60 kDa) in purified histone H1 from the calf thymus and identified it as a histone H1 by ESI-MS/MS analysis (data not shown). Further studies, including an investigation of the localization of histone H1 and its translocation from the nucleus to the cytoplasm in the course of allergen-IgE cross-linking, should be performed to clarify the fundamental mechanisms of mast cell activation and degranulation by nuclear histone H1. The target receptors for histone H1 and underlying signal transduction are also currently under investigation.

In addition to the pathophysiological roles of histone H1 on mast cell-mediated type I allergic responses, we have demonstrated the therapeutic potential of SSV mAb, which targets the histone H1 in allergic inflammation (Figs 4 and 5). Because SSV mAb does not affect OVA-specific immunoglobulins (Fig 6), the most probable mechanism behind the inhibition of type I allergic responses may be a physical blockage of histone H1, which nonspecifically induces the degranulation of IgE-binding mast cells. Therapeutic potential of SSV mAb via the suppression of plasma and lung levels of histone H1 has been recently demonstrated in a mouse model of LPS-induced sepsis-like syndrome [14]. In this study, we could not confirm the neutralizing effect of SSV mAb on the circulating histone H1 level (Fig 8A). However, SSV mAb significantly and successfully suppressed the local expression of histone H1 in the nasal mucosa (Fig 8B), suggesting that this anti-histone H1 mAb fulfilled anti-allergic potency by specific recognition of local histone H1. Another potential mechanism behind the inhibition of type I hyperreactivity by SSV mAb is the modification of the immune cell cytokine profile. In our *in vivo* study, the administration of SSV mAb slightly activates IL-5- and IL-13-producing cells (Fig 7). The production of IL-5 and IL-13 is a hallmark of both pathogenic allergic inflammation and protective anti-helminth parasitic immune responses at mucosal sites [38]. Due to the undetermined nature of target cells for SSV mAb and its recognized cell-surface molecules, it is difficult to understand the precise mechanisms by which SSV mAb leads to the inhibition of type I allergic responses. A previous study mentioned that extracellular histones trigger toll-like receptor (TLR)2 and TLR4 signaling in the models of Concanavalin A-induced sterile inflammation and acetaminophen-induced hepatotoxicity [39]. Our pilot study using hepatic stellate cells indicated the elevation of TLR4 by histone H1 (unpublished data). Further studies, including the characterization of target cells/molecules for SSV mAb and its down-stream signal transduction, should be performed to understand the histone H1-mediated type I allergic responses and their suppression by SSV mAb.

In summary, our present data demonstrated that linker histone H1, which is released not only from damaged cells but also from activated mast cells, nonspecifically induced mast cell degranulation of IgE-coated mast cells both *in vitro* and *in vivo*. Furthermore, exogenous histone H1 significantly enhanced DNP-BSA-induced mast cell degranulation. These results strongly suggest the impact of endogenous histone H1, which may act as an alarmin, on the enhancement of allergen-independent exacerbation of type I hyperreactivity. In the clinical setting, the elevation of anti-nuclear Abs has been reported in patients with chronic rhinosinusitis [40]. We need to clarify the balance of nuclear antigens and corresponding Abs in patients with type I hyperreactivity to evaluate the functional meaning of these phenomena.

## Supporting Information

S1 Fig Induction of total IgE but not anti-histone H1 IgE by OVA/alum sensitization. (DOCX)

S2 Fig Induction of proinflammatory cytokines in the course of degranulation. (DOCX)

S1 Protocol (DOCX)
