# Supplementary material for: Impact of Histone H1 on the Progression of Allergic Rhinitis and Its Suppression by Neutralizing Antibody in Mice
Source: PLoS One. 2016 Apr 18;11(4):e0153630. doi: 10.1371/journal.pone.0153630 (PMC4835108; doi:10.1371/journal.pone.0153630)
Supplement: S1 Fig — (DOCX) [file pone.0153630.s001.docx]

**S1 Fig. Induction of total IgE but not anti-histone H1 IgE by OVA/alum sensitization.** Total and histone H1-specific IgE levels were measured by ELISA. **, *P*<0.01 versus the pre-immunized serum. NS: not significant.
