# Supplementary material for: Impact of Histone H1 on the Progression of Allergic Rhinitis and Its Suppression by Neutralizing Antibody in Mice
Source: PLoS One. 2016 Apr 18;11(4):e0153630. doi: 10.1371/journal.pone.0153630 (PMC4835108; doi:10.1371/journal.pone.0153630)
Supplement: S2 Fig — (DOCX) [file pone.0153630.s002.docx]

**Figure S2. Induction of proinflammatory cytokines in the course of degranulation.** Exogenous histone H1 (100 μg/ml) strongly induced proinflammatory cytokine release as compared with IgE-antigen (Ag) cross-linking. Data are represented as the mean ± S.D. **, *P*<0.01 versus (IgE + Ag).
