# Supplementary material for: Impact of Histone H1 on the Progression of Allergic Rhinitis and Its Suppression by Neutralizing Antibody in Mice
Source: PLoS One. 2016 Apr 18;11(4):e0153630. doi: 10.1371/journal.pone.0153630 (PMC4835108; doi:10.1371/journal.pone.0153630)
Supplement: S1 Protocol — (DOCX) [file pone.0153630.s003.docx]

**Supporting information for**

Impact of histone H1 on the progression of allergic rhinitis and its suppression by neutralizing antibody in mice

Toshiaki Nakano^1,2^*, Rikiya Kamei^3^, Takashi Fujimura^3^, Yuki Takaoka^2,4^, Ayane Hori^3^, Chia-Yun Lai^2^, Kuei-Chen Chiang^5^, Yayoi Shimada^5^, Naoya Ohmori^5,6^, Takeshi Goto^5,6^, Kazuhisa Ono^3,7^, Chao-Long Chen^2^, Shigeru Goto^2,6,8^, Seiji Kawamoto^3^*

**S1 Protocol**

*Total IgE ELISA*

To evaluate the total IgE level, anti-mouse IgE (250× dilution with 100 mM NaHCO_3_ (pH 8.5); BD Biosciences San Jose, CA, USA) was coated onto 96-well microtiter plates (Nalgene Nunc International, Roskilde, Denmark) by overnight incubation at 4 °C. The plate was then blocked with 1% (w/v) bovine serum albumin (BSA)/PBS for 2 hrs at 37°C, and serum samples (10× dilution with blocking buffer) were added to each well and incubated for 2 hrs at 37°C. Next, biotin-conjugated anti-mouse IgE (250× dilution; BD Biosciences) was added and incubated at 37°C for 1.5 hrs. Streptavidin-HRP (200× dilution; R&D Systems, Minneapolis, MN, USA) was then added and incubated at 37°C for 1 hr, followed by the addition of 1-Step Ultra TMB substrate solution (Thermo Fisher Scientific Inc., Rockford, IL, USA). The absorbance at 450 nm was then measured using a Victor X4 Multilabel Plate Reader (PerkinElmer, Waltham, MA, USA).

*Anti-histone H1 IgE ELISA*

To evaluate the anti-histone H1 IgE titer, 2 μg/ml of calf thymus histone H1 (Millipore, Billerica, MA, USA) in 100 mM NaHCO_3_ (pH 9.3) was coated onto a 96-well microtiter plates (Nalgene Nunc International) by incubation at room temperature for 1 hr. The plate was then blocked with SuperBlock T20 (PBS) Blocking Buffer (Thermo Fisher Scientific Inc.), and serum samples (50 μl, 100× dilution with 10 mM Tris-HCl (pH 8.0), 0.9% (w/v) NaCl, 0.5% (w/v) Tween 20) were added to the wells. The mixture was incubated at room temperature for 1 hr, followed by the incubation with HRP-conjugated anti-mouse IgE (2000× dilution; Bethyl Laboratories, Inc., Montgomery, TX, USA) for 1 hr. ABTS substrate solution (Sigma, St. Louis, MO, USA) was added for color development and the absorbance at 405 nm was then measured using a Victor X4 Multilabel Plate Reader (PerkinElmer).

*Cytokine ELISA*

The supernatant of RBL-2H3 cells after IgE-antigen cross-linking or histone H1 (100 mg/ml) treatment were collected, and IL-6 and TNF-α levels were measured using Rat IL-6 and TNF-α ABTS ELISA Development Kits (PeproTech, Rocky Hill, NJ, USA), according to the manufacturer’s protocols.
